# Migratory Connectivity of Zhejiang, with a Critical Stopover in East Asian-Australasian Flyway, Based on Recovery Data

**DOI:** 10.3390/ani14162404

**Published:** 2024-08-19

**Authors:** Baoquan Liu, Hongdi Gao, Jinhui Wang, Zhenxian Zhu, Cheng Qian, Zhongyong Fan, Ke He

**Affiliations:** 1 College of Animal Science and Technology College of Veterinary Medicine, Zhejiang Agriculture and Forestry University, Hangzhou 311300, China; 2 Zhejiang Forest Resource Monitoring Center, Hangzhou 310020, China; zjhzlbq@126.com (B.L.);; 3 Zhejiang Museum of Natural History, Hangzhou 310012, China

**Keywords:** recovery of bird-ringing, waterbirds, migratory connectivity, EAAF, Zhejiang coast

## Abstract

**Simple Summary:**

Using a combination of recapture records from bird-ringing projects and community science re-sighting data, we evaluated 208 records representing 35 species. The connectivity between the Zhejiang coast and various countries (i.e., Russia, Mongolia, the USA, Korea, Japan, Malaysia, Singapore, Thailand, and Australia) and regions within China was established. Furthermore, we identified areas that should be prioritized in conservation efforts, including unprotected coastal wetlands along the Wenzhou coastline. These results provide important insight into vulnerable migratory waterbirds.

**Abstract:**

Understanding migratory routes is crucial for the conservation of birds and their habitats. Zhejiang is a crucial stopover and wintering area for birds in the East Asian–Australasian Flyway; however, detailed information on this area, and particularly on connections between coastal areas, is limited. By synthesizing ringed and recapture records from local bird-ringing projects and re-sighting community science data (208 records of 35 species), we established migratory connectivity between the Zhejiang coast and nine countries (i.e., Russia, Mongolia, the United States, Korea, Japan, Malaysia, Singapore, Thailand, and Australia), as well as eleven sites within China, and established its crucial role in this flyway. Stopover fidelity was verified by some species with high recapture frequency (seven species exceeded 1%) and species with duplicated re-sighted records (seven Black-faced Spoonbill, one Dalmatian Pelican, and two Spoon-billed Sandpiper individuals). We identified six areas—Hangzhou Bay, Aiwan Bay, Xuanmen National Park, Wenzhou Bay, the reclaimed area between the Ou and Feiyun Rivers, and the Wenzhou Jiangnan Reclamation Area—as crucial stopovers and wintering refuges for waterbirds. Notably, in Xuanmen National Park and the coastal regions along Wenzhou, there were many recovery records for flagship species, such as the Black-faced Spoonbill and Spoon-billed Sandpiper. There were several cases of the recovery of the same individual studied across the years. These findings indicate that these unprotected wetlands require particular attention. Broadly, our findings highlight the feasibility of integrating comprehensive ringing projects with citizen science data to formulate effective conservation strategies and underscore the critical importance of the Zhejiang Coast for migratory waterbirds, particularly those with high conservation concerns, emphasizing the need to mitigate the threats faced by these vulnerable populations.

## 1. Introduction

Global climate change has affected the distribution and migration of many bird species (the 2022 World Bird Status Report by BirdLife International). Populations using different habitats may experience different levels of pressures, causing discrepant survival of populations, such as the whimbrel (*Numenius phaeopus*) in Australia; furthermore, different population trends have been discovered in Moreton Bay and Roebuck Bay populations [1]. Migratory birds have geographically complex life cycles, with breeding, stopover, and non-breeding sites distributed across vast areas, making them susceptible to threats along their migratory routes [2,3]. Consequently, establishing a network of protected areas is crucial for conservation efforts; however, this endeavor requires comprehensive data on population dynamics and migration patterns, underscoring the significance of avian monitoring initiatives.

China has three major migratory flyways, as follows: the East Asia–Australia Flyway (EAAF), the Central Asia–India Flyway, and the West Pacific Flyway. The EAAF is renowned for its diverse bird population, consisting of 600 species (https://eaaflyway.net/, accessed on 1 July 2024). Moreover, the EAAF intersects with the West Pacific Flyway. The Central Asian Flyway supports a rich variety of waterbirds, raptors, landbirds, and seabirds [4]. As the most densely populated flyway in the world, the EAAF provides a habitat for nearly 2 billion people, and serves as a migratory route for 28 to 68 million waterbirds [5]. Priority coastal wetlands of EAAF for waterbirds have been identified in China [6,7] and Australia [8]. Studies have revealed declines in the populations of many migratory waterbird species within the Yellow Sea tidal zone [9,10], but have not provided an explanation for this phenomenon. Extensive research and monitoring efforts have focused on the ecological significance of the Beibu Gulf [11], South China Coastal Wetlands [12], Deep Bay [13], and Tiaozini Wetland [14] for migratory waterbirds; however, the connections between the southeastern coasts of China are not clearly established.

Zhejiang, located on the southeastern coast of China, plays a pivotal role in the conservation of migratory waterbirds in the EAAF [6,7], and includes extensive tidal wetlands along several coastlines. In annual surveys conducted from 2017 to 2024, nearly 100,000 wintering waterbirds were recorded in Zhejiang. Despite its ecological significance, research on migratory waterbirds in this EAAF region remains limited. For example, during the migration of whimbrel, several important stopover sites were identified, including Chongming in the Yellow Sea [1,15,16]. Our field survey of Zhejiang’s coast also functions as an energy-refueling site for this species (synchronous survey data of wintering waterbirds since 2017), but this is not reported. Populations of Nordmann’s Greenshank (*Tringa guttifer*) were considered to represent more than 10% of the global population in Shangdong, Hebei, and Jiangsu [17], and the species was recorded in Zhejiang during a bird-ringing project. Notably, the endangered Black-faced Spoonbill (*Platalea minor*) and Spoon-billed Sandpiper (*Calidris pygmaea*) were observed at stopover sites in Wenzhou Bay [18]. However, this southeast coastal wetland area on the migration route is understudied, and there is a knowledge gap regarding connectivity.

In addition to the annual monitoring of waterbirds, in Zhjiang, regular bird-ringing activities have been conducted, and citizen bird-watching is increasing. Recapture records of banded birds have provided valuable insights into the dynamics of various avian species. These insights have enabled the estimation of population sizes and survival rates [19], and serve as evidence for understanding migratory routes, stopover sites, wintering areas [20], and subspecies differentiation (e.g., in studies of the Bar-tailed Godwit [*Limosa lapponica*] populations by Wilson et al., 2007 [21]). Furthermore, these long-term ringing records play a crucial role in decision-making regarding animal conservation strategies and habitat protection. For example, recovery records for the Black-necked Crane (*Grus nigricollis*) revealed three migration routes, providing guidance for conservation efforts [22]. Furthermore, citizen-supported field observations offer invaluable opportunities for gathering information about the distribution and abundance of migratory birds across different habitats, such as waterbirds [23,24], or even elusive landbird species [25]. In Zhejiang, these endeavors provide opportunities to explore the connections between other regions, and guide effective conservation actions.

In this study, we used bird-ring records and ring-recovery records (including those recaptured from bird-ringing projects conducted between 2018 and 2024, and re-sighted birds with regular monitoring work from 2021 to 2024) to achieve the following three aims: (1) assess the species composition and abundance fluctuation of the bird-ringing project; (2) identify important and consistent stopover sites for migratory birds in Zhejiang based on recovered records; and (3) evaluate the migratory connectivity of Zhejiang’s coasts within the EAAF.

## 2. Material and Methods

### 2.1. Study Area

The bird-ringing project was undertaken in Hangzhou Bay (HZW, 29°58′27″ to 30°51′30″ N and 120°54′30″ to 121°50′48″ E), a typical coastal wetland ecosystem comprising coastal mudflats, shallow sea areas, and reclaimed wetlands such as intertidal salt marshes and aquaculture farms. Birds were captured using mist nets in the tidal zone, and were banded. The captured birds were recorded and banded at their respective stations before release. More than 3000 individuals were banded.

The daily monitoring encompassed multiple regions across Zhejiang Province. The study area in Zhejiang is on the southeastern coast of China and encompasses several bays and estuaries with extensive mudflats. This region has many rivers, including the Yong, Jiao, Ou, Feiyun, and Ao Rivers, which flow into the Hangzhou Bay, Wenzhou Bay, and Yueqing–Xuanmen Bay. Monitoring was conducted by the Zhejiang Bird Watching Society (2021 to 2024). Various marking methods, such as metal rings, wing tags, neck bands, leg rings, and flags, along with telemetry equipment, have been employed. The selection of ringing sites used the EAAF color-flagging protocol, in addition to information exchanged with the China National Bird Ringing Center and communication with local and international bird research teams.

### 2.2. Data Sources

From 2018 to 2024, bird-ringing projects were conducted in April and May (spring, 2018 to 2019, and 2022 to 2024), and in November (autumn, 2020 and 2021).

The recovery data were derived from the following two sources: recapture data from local bird-ringing projects, and re-sighting data obtained during daily monitoring, with both including locally banded birds, as well as those banded at other sites (Table 1). Based on location data for marked individuals and recovery records, two sets of recovery records were compiled for this study, as follows: (I) recaptured individuals from HZW during local bird-ringing projects, including (I-a) locally banded birds and (I-b) those banded at other sites, and (II) re-sighted individuals from (II-a) local bird-ringing projects and (II-b) off-site locations within Zhejiang during daily monitoring.

### 2.3. Data Analysis

#### 2.3.1. Composition of Ringing Birds and Recovered Species in Zhejiang

The bird-ringing records in HZW (dataset I) were used to calculate the composition and frequency of migratory birds in spring. Only banded waterbirds were analyzed for their abundance. The top ten species of each year form a list of commonly observed species. Because the total number of ringed individuals varied between years, we also calculated the frequency of the top ten species in each year. To assess the consistency of waterbirds between years, the abundance and frequency of their occurrence between years were analyzed and visualized using the “ggplot” package in R (version 3.6.3). We employed a generalized linear mixed model (GLMM) to analyze the count records of commonly observed species, considering seasons or years as fixed effects, and species as a random intercept. This approach allowed us to account for the variability in record counts across seasons or years, and to consider the nested structure of species within the data. The model was fitted using the “glmer” function from the “lme4” package in R. The statistical significance of the fixed effects in the model was assessed using analysis of variance (ANOVA), with the “Anova” function in the “car” package in R.

Data from (II) were used to analyze the species, abundance, and frequency of re-sighting in Zhejiang. Migratory behavior was classified into the following modes: northward migration for breeding purposes, southward migration for wintering, resident birds that do not migrate, breeding birds that stay within their habitat year-round, and wintering birds that temporarily reside outside of their breeding range during the cold months.

#### 2.3.2. Migration Connectivity

Connectivity between the Zhejiang coast and other regions was evaluated using data from (I-b) and (II-b), excluding non-traceable records, such as individuals with trackers but no information about their origin. Migration connectivity was studied by conducting an initial analysis of Zhejiang Province. The following sites in China were merged based on geographical region: Jiangsu included Rudong and Dongtai; Guangdong–Hong Kong included Shenzhen Bay and the Mai Po Nature Reserve; and Bohai Bay included Nanbao Salt Lake in Tangshan, the Liaohekou Nature Reserve in Panjin, the Yellow River Delta in Dongying, and Tanggu in Tianjin. Records from outside of China were categorized by country, except for four regions in Australia (Northwestern Australia [NWA], Northern Territory, South Australia [SA], and Victoria) and two regions in Russia (Kamchatka and Chunkotka). Species with recovery records originating from three or more areas can provide valuable insights into migration routes. The data for the breeding, non-breeding, and stopover sites of these species (Great Knot [*Calidris tenuirostris*], Dunlin [*Calidris alpine*], Red Knot [*Calidris canutus*], Red-necked Stint [*Calidris ruficollis*], and Terek Sandpiper [*Xenus cinereus*]) were from existing studies and the IOC World Bird List [26,27].

Migration connectivity was investigated in critical wetlands to identify priority sites that require special attention (including re-sighting records of locally or internationally banded individuals and recapture records of international individuals; datasets I-b and II). Sites with fewer than 10 records were excluded from the analysis. Based on their location, the remaining sites were classified into six areas, as follows: HZW, Xuanmen Bay (XMW), Aiwan Bay (AWW), Wenzhou Bay (WZW), the reclaimed area between the Ou and Feiyun Rivers (OJ-FYJ), and the Wenzhou Jiangnan Reclamation Area (JNR). Results were visualized using ArcGIS Pro (Esri, Inc., Redlands, CA, USA) and GraphPad Prism 10.1.2 (Boston, MA, USA).

## 3. Results

### 3.1. Species Composition and Abundance of the Bird-Ringing Project in Recent Years

**Figure 1:**
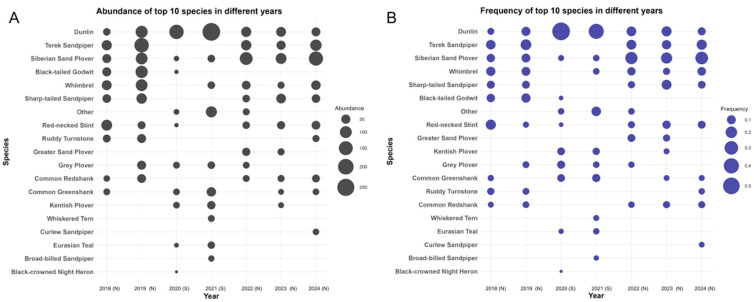
(**A**) Abundance and (**B**) frequency of the top 10 species in different years of HZW’s bird-ringing project. N: northward migration (April and May); S: southward migration (October and November).

Sixty-three species were recorded during the bird-ringing project in HZW, and most of these birds were waders, such as Dunlin, Siberian Sand Plover (*Charadrius mongolus*), Whimbrel, and Terek Sandpiper (Table S1, Figure 1).

Some differences were found between seasons, but not between years. When considering the season and year effect, the results from the GLMM and subsequent ANOVA suggested that the count records of species were largely consistent across the spring seasons, except for 2019 (*p* < 0.001 when 2019 vs. other years). The increase in banded individuals in 2019 might have contributed to the observed significance, and further analysis using species frequency did not show significant differences (ANOVA year *p* > 0.05). In the seasonal analysis, a significant difference was observed between spring and summer (ANOVA season *p* < 0.001, spring vs. fall *p* < 0.001). This result indicated distinct migration behaviors during these two seasons that affect the count records of species. Certain Siberian Sand Plover, Sharp-tailed Sandpiper (*Calidris acuminata*), Red-necked Stint, and Terek Sandpiper populations in the northward and southward periods used HZW as a stopover, and the individual abundance in spring was higher than that in fall. The records of Dunlin showed the opposite phenomenon (Figure 1). The maintenance of a relatively stable species community implied that HZW is an important stopover for waterbird migration.

### 3.2. Migratory Stopover Frequency, Supported by Recaptured Birds

**Figure 2:**
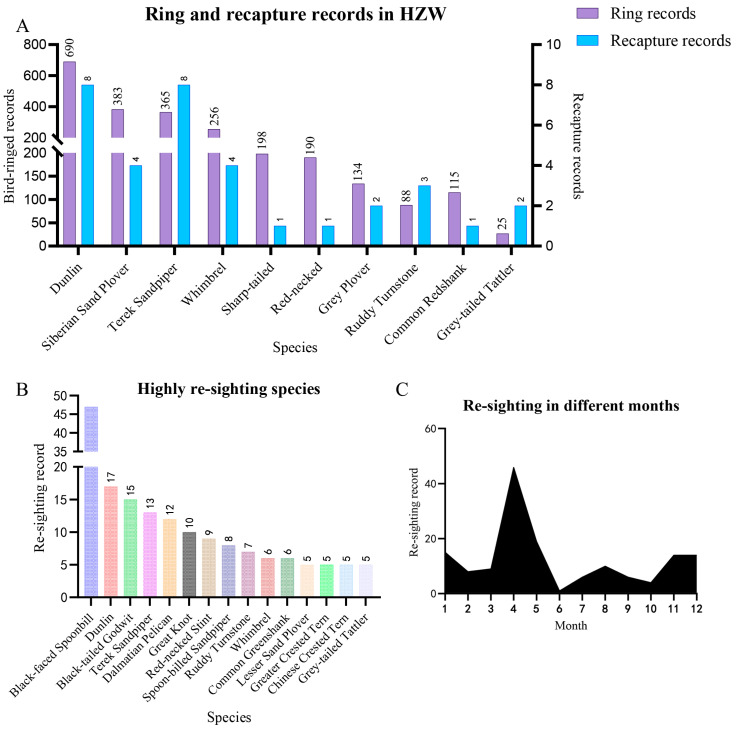
The recapture and re-sighting records in Zhejiang Province. (**A**) The ring and recapture records in HZW. (**B**) The species with high-frequency recordings (including recaptured and re-sighting) in Zhejiang Province. (**C**) The temporal distribution of record numbers was analyzed for different months.

A total of 34 individual data points and 35 re-caught records were collected using mist netting in HZW (2018 to 2024, dataset I). Based on the cumulative recapture numbers during this period, seven species had a recapture frequency that exceeded 1%, except for the following three waders: Common Redshank (*Tringa totanus*), Red-necked Stint, and Sharp-tailed Sandpiper (Figure 2A). The high recapture ratio of 7.4% for the Grey-tailed Tattler (*Tringa brevipes*) might be overestimated because of the low record number of this species. The high recapture frequencies of the species suggested they regularly use this wetland stopover to refuel during migration.

### 3.3. Combing Recovery Records Revealed the Migration Connection between Zhejiang and Other Regions in the EAAF

#### 3.3.1. Recovery Records Suggested Differences between Migratory Periods in Zhejiang

During daily monitoring in Zhejiang Province, 148 records of non-indigenous banded individuals were documented from 2021 to 2024, representing 25 species and 129 individuals (dataset II-b, Table S2 also included 19 local banded individuals). All re-caught and re-sighting records were combined as the recovery data (datasets I and II), including 34 species, 189 individuals, and 209 records. The number of recovery records was highest for Black-faced Spoonbill (47 sightings and 36 individuals), followed by Dunlin, Black-tailed Godwit (*Limosa limosa*), Terek Sandpiper, Dalmatian Pelican (*Pelecanus crispus*), and Great Knots, all with more than 10 re-sightings each (Figure 2B). Noteworthy recurrent re-sightings included the following: seven individual Black-faced Spoonbill observed two or three times within nearly one to two years, four recorded instances of a single Dalmatian Pelican within one to nine months, two individuals spotted twice and thrice for Spoon-billed Sandpiper within one to five months, and three for Curlew Sandpiper (*Calidris ferruginea*) within three to seven months. The high re-sighting frequencies for some species might be explained by their endangered status (Black-faced Spoonbill, Dalmatian Pelican, and Spoon-billed Sandpiper), body size (Black-faced Spoonbill and Dalmatian Pelican), and population abundance (Dunlin and Red-necked Stint). In field bird-watching, the species the public were attracted to tended to be flagship species, because of their rarity and striking characteristics. A large grouping of individuals tends to capture the attention of bird watchers more effectively, and these concentrations facilitate the detection of marked individuals within the population.

The recovery frequencies were high during the spring (April and May, corresponding to the northward migration of major bird species) and winter (November to March, associated with southward migration or wintering) (Figure 2C). For the northward migration, the recovery records accounted for 58.05% of the total, with a continuous increase from 2022 to 2024.

#### 3.3.2. Migratory Connectivity between Zhejiang and Other Regions in the EAAF

**Figure 3:**
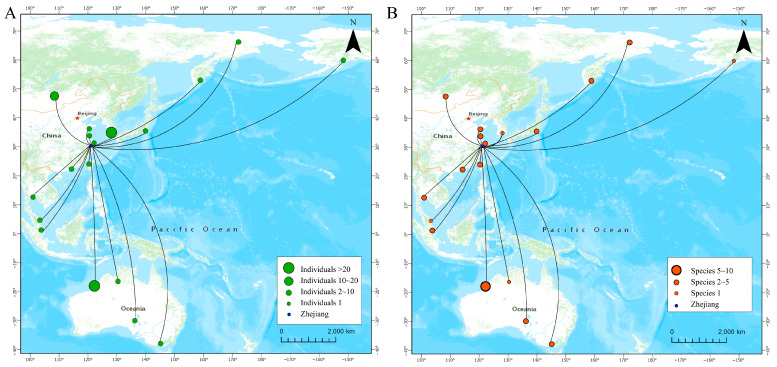
Migratory connectivity of marked waterbirds in Zhejiang based on (**A**) individuals and (**B**) species. The size of the dots indicated the number of birds observed.

Waterbirds in the coastal wetlands of Zhejiang (datasets I-b and II-b) are connected to at least eighteen locations in the EAAF (Figure 3), as follows: four regions in Australia, two regions in Russia, five regions in China, and seven other countries (i.e., Japan, Thailand, the United States, Malaysia, Singapore, Korea, and Mongolia). The number of occurrence records was highest in Australia (forty occurrences and fourteen species) and Korea (forty-three occurrences of one species), followed by Mongolia (twelve occurrences and four species) and Russia (nine occurrences involving six species). In the remaining regions, five or fewer occurrences were recorded (Figure 3A). In mainland China, connections were detected between Zhejiang and some stopover sites, including Jiangsu, the Chongming Islands in Shanghai, Guangdong–Hong Kong, and Bohai Bay.

**Figure 4:**
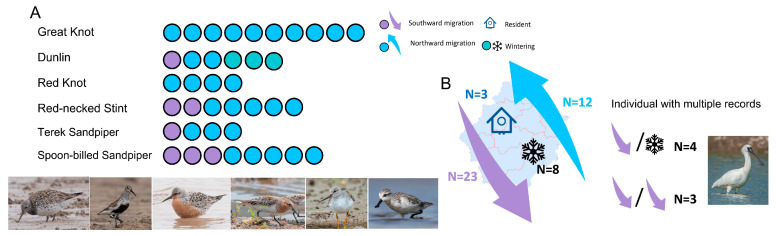
(**A**) The recovery species with a source of more than three regions, and the different migratory behaviors of their records. (**B**) The migratory behaviors of Black-faced Spoonbill’s records.

The recovery records of most species were concentrated in one or two regions. However, for six species, the recovery records originated from three or more areas (Table 2). Among these species, five of them (except the Terek Sandpiper) supported Zhejiang’s migratory connections between breeding sites and non-breeding sites. All of the breeding sites were in Russia, and the non-breeding sites included Australia, Singapore, and Thailand. All of the species’ records included the northward migration. Conversely, southward migration was not recorded in Great Knot and Red Knot (Figure 4A). As the species with the highest recorded frequency, Black-faced Spoonbill was banded at limited sites, banded as chicks on the Chilsando Islet (one site, one individual) or Gyeonggi Bay (thirteen sites with thirty individuals), and adults were rescued in Taiwan, China, which had the non-breeding sites. The re-sighted records of Black-faced Spoonbill covered all periods, including northward migration, southward migration, wintering, and resident (Figure 4B).

### 3.4. Important Migratory Bird Monitoring Sites in Zhejiang

**Figure 5:**
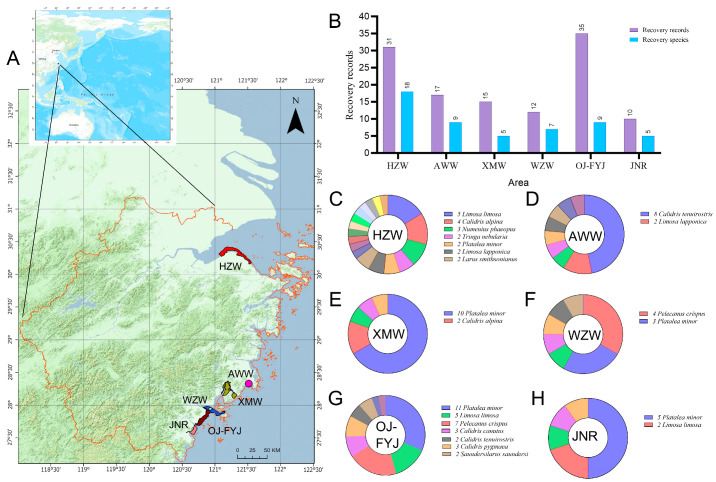
The hotspot of banded bird recovery in Zhejiang. (**A**) Location of the six critical coastal wetlands; (**B**) comprehensive records and species diversity found within these six sites; and (**C**–**H**) detailed information on species abundance of recovery records in each site, with labeled names assigned only to those species observed more than once.

The recovery records of ringed birds (dataset II), varied among the monitoring sites in Zhejiang, with six sites reporting counts more than ten times higher than those at other sites (Figure 5). Among these sites, OJ-FYJ had the most records (thirty-five, accounting for 20.11% of the total recovery records), and nine species (31.03%). HZW had the highest species diversity, with 18 species, and a similarly high probability of recovering marked birds (31 events, 17.82%). Other areas showed lower rates of recovery (ten to seventeen events) and species diversity (five to nine species) than OJ-FYJ and HZW (Figure 5B).

Based on multiple recovery records and the endangered status of the Black-faced Spoonbill, Spoon-billed Sandpiper, and Dalmatian Pelican, we analyzed the conservation role of coastal wetlands in Zhejiang. Four sites in south Zhejiang, along the Wenzhou and Taizhou coasts, showed a high frequency of recovery for Black-faced Spoonbills (Figure 5E–G), and the Wenzhou coast was the preferred wintering ground for the Dalmatian Pelican (Figure 5F,G). For Spoon-billed Sandpiper species, five individuals with eight records were observed, with connections between the Chunkotka and Kamchatka regions in Russia, and Thailand. The sites hosting these flagship species included XMW, OJ-FYJ, and Zhanghua Bay, and should be researched because they are not listed among the six important monitoring regions, despite the continuous records for three years involving a single individual (ID: Orange A6).

## 4. Discussion

By synthesizing recapture records from local bird-ringing projects and re-sighting community science data along the Zhejiang coast, we established migratory connections between the Zhejiang coast and nine countries and eleven sites within China, which indicated Zhejiang’s indispensable role in EAAF. Waders show a high site fidelity to this region, as is evident from the high recaptured frequency of several waterbirds; the fidelity was more important in northward migration than in southward migration, evidenced by a significant difference in re-sighted records across Zhejiang, and bird-ringing records in HZW. We also found that six areas (HZW, AWW, XMW, WZW, OJ-FYJ, and JNR) might be crucial stopovers and wintering refuges for waterbirds. Notably, recovery records were high in XWM and the coastal regions along Wenzhou for flagship species such as Black-faced Spoonbills and Spoon-billed Sandpipers. Therefore, unprotected wetlands require further research.

### 4.1. Zhejiang Has a Pivotal Role in the EAAF

Coastal areas along the EAAF play a paramount role in supporting migratory birds because they serve as crucial stopover sites for refueling and replenishing energy reserves. However, safeguarding only a few key sites is insufficient to ensure an effective conservation “safety net” for avian species [28]. Therefore, establishing an interconnected network of sites within this flyway is imperative to conserve migratory waterbirds. Tracking studies have revealed migration strategies and flyways of large-bodied birds in Asia [29,30,31,32]. However, owing to the high cost of telemetry equipment, tracking efforts are limited in species coverage and individual representation. Bird-ringing is a cost-effective, reliable method for studying migration patterns. In our study, local and off-site recovery records strongly indicated that the coast of Zhejiang served as a vital refuge for the migration of waterbirds within the EAAF. Furthermore, synchronized surveys conducted during the winter identified over 100,000 individuals belonging to 86 different waterbird species in 2024, underscoring the importance of Zhejiang’s coast for these migratory waterbirds.

Bird-ringing efforts initiated in Chongming in 1979 have yielded significant insights. Recaptured individuals from 17 countries and regions provided valuable data for understanding the complex patterns of bird migration through citizen science [33]. However, identifying countries and regions with low recapture percentages within a short timeframe can be challenging. For example, our dataset did not include individuals from Tasmania of Australia, probably because of their infrequent migratory paths intersecting our study area. This observation aligns with that of existing research, indicating that detecting less common migratory routes requires more prolonged and extensive monitoring than usual ones [34]. The high recovery rate observed in the Beibu Gulf underscores its critical importance as a stopover site for the Spoon-billed Sandpiper [35], a species of significant conservation concern. This finding is consistent with an existing study highlighting the Beibu Gulf as vital for the survival of various migratory shorebirds [11]. Such data can inform conservation strategies and provide a basis for the protection of these key habitats. Additionally, species exhibiting high frequencies of recovery (Figure 2C) highlight the pivotal role of Zhejiang in their migratory journeys. The abundance of recoveries in this area provides valuable insights into the migratory behavior and habitat utilization of these species, contributing to the understanding of their ecological requirements.

### 4.2. Conservation of Black-Faced Spoonbill and Spoon-Billed Sandpiper

Black-faced Spoonbill is the rarest species in the genus *Platalea*. Its breeding habitat is located in the northern Yellow Sea [32,36], and it winters along the East Asian coast as far as the Red River in Vietnam [37]. In 2023, its population reached a new record high of 6633 individuals (by the Hong Kong Bird-Watching Society). Synchronized international surveys were conducted for this species at 42 wintering sites between 1997 and 2014 [38]. However, this latest study included few coastal sites in southern China, leaving a gap between the Yangtze Estuary (Chongming in Shanghai) and Fujian Province. The tracking results suggested that HZW and Taizhou Bay in Zhejiang serve as wintering areas for this species, and a revised distribution map of Black-faced Spoonbills indicates the inclusion of the Zhejiang coast as a staging area [32,39]. Although Zhejiang has been included in the census report of the Hong Kong Bird-Watching Society, detailed information is necessary. Our recovery data revealed approximately 15 to 19 ringed individuals with records of stopovers or wintering in Zhejiang, connected to Korea and Taiwan, two major breeding and wintering sites for this population. According to synchronized surveys of wintering waterbirds over the past five years, approximately 142 to 215 individuals have been observed during the wintering period in Zhejiang. Notably, XMW is considered a key site for wintering and stopovers. Based on continuous monitoring at XMW from 2014 to 2017 during the winter period, the maximum single-day count was 86 [40], and the average number of individuals observed from 2015 to 2021 exceeded 1% of the global population (3356 to 5222) [41]. Therefore, we strongly recommend the inclusion of the Wenzhou coastline, such as WZW, OJ-FYJ, and JNR, as significant observation sites for this avian species. Black-faced Spoonbills are sensitive to human disturbances [42]. Because of insufficient protective measures in these areas, implementing long-term conservation plans and habitat management strategies to safeguard fishponds, mudflats, and coastal wetlands in this region is imperative.

Spoon-billed Sandpiper is a critically endangered shorebird species that migrates exclusively within the EAAF; breeds in the Russian Arctic during the boreal summer; and undertakes extensive migrations of thousands of kilometers to overwinter in southern China, Southeast Asia, and South Asia. The species showed a rapid population decline in the past decades [43,44], with an average annual rate of decline of 8% from 2014 to 2019 [45]. Evidence suggests that hunting activities in wintering areas [46] and habitat loss at staging and stopover sites contribute to this decline [47]. Large-scale land claims, degradation of intertidal mudflat habitats due to the spread of invasive *Spartina* [47], and entanglement in nets pose serious threats to the survival of this species [48]. Jiangsu Province, which is adjacent to Zhejiang, serves as a crucial stopover and molting site for Spoon-billed Sandpiper species [17,49]. Tiaozini, Yangkou, and Dongling were the three primary sites for this purpose [50]. In contrast with Jiangsu Province, where information on this species is relatively abundant, there is limited documentation of its presence in Zhejiang [44]. Unlike the Black-faced Spoonbill, there are no records of Spoon-billed Sandpiper during synchronized surveys of wintering waterbirds. An explanation might be that the survey periods did not coincide with southward or northward migration periods. However, considering the high habitat specificity of Spoon-billed Sandpipers, multiple sightings of individuals in Zhanghua Bay (Orange A6) and on the Ruian coast (Orange K9) suggest that these tidal areas provide suitable habitats. Further research on this species should be expanded to nearby regions.

## 5. Conclusions

This study represents the first analysis of waterbird migration along the Zhejiang Coast that utilized local bird-ringing and community science data. However, our study yielded a relatively low recovery rate compared to those of synchronized surveys of wintering waterbirds in Zhejiang and continuous monitoring at certain sites. Despite ongoing bird-ringing projects in HZW, as well as the growing availability of citizen science data for Zhejiang, the vast expanse of intertidal flats spanning 2218 km along the Zhejiang coast necessitates an increase in the number of observers. Additionally, we observed that bird watchers tended to focus on specific flagship species, potentially limiting data collection for other species. In further research, satellite tracking should be employed to comprehensively elucidate bird migration patterns, particularly for high-priority species.

## Figures and Tables

**Table 1:** Recovery records of banded bird species from Zhejiang coast (2018 to 2024).

Order	Family	Species	Common Name	National Priority Protection in China	IUCN Red List	Records	
						Recapture Frequency	Re-Sighting Frequency
Anseriformes	Anatidae	*Anser albifrons*	White-fronted Goose	II	LC		2
Anseriformes	Anatidae	*Anser serrirostris*	Tundra Bean Goose		-		1
Anseriformes	Anatidae	*Cygnus columbianus*	Tundra Swan	II	LC		2
Ciconiiformes	Ciconiidae	*Ciconia boyciana*	Oriental Stork	I	EN		2
Charadriiformes	Recurvirostridae	*Recurvirostra avosetta*	Pied Avocet		LC		3
Charadriiformes	Charadriidae	*Charadrius mongolus*	Lesser Sand Plover		EN	4	1
Charadriiformes	Charadriidae	*Pluvialis squatarola*	Grey Plover		LC	2	1
Charadriiformes	Laridae	*Larus crassirostris*	Black-tailed Gull		LC		1
Charadriiformes	Laridae	*Larus smithsonianus*	Vega Gull		LC		3
Charadriiformes	Laridae	*Saundersilarus saundersi*	Saunders’s Gull	I	VU		3
Charadriiformes	Laridae	*Thalasseus bergii*	Greater Crested Tern	II	LC		5
Charadriiformes	Laridae	*Thalasseus bernsteini*	Chinese Crested Tern	I	CR		5
Charadriiformes	Scolopacidae	*Arenaria interpres*	Ruddy Turnstone	II	LC	3	4
Charadriiformes	Scolopacidae	*Calidris acuminata*	Sharp-tailed Sandpiper		LC	1	
Charadriiformes	Scolopacidae	*Calidris alpina*	Dunlin		LC	8	9
Charadriiformes	Scolopacidae	*Calidris canutus*	Red Knot		NT		4
Charadriiformes	Scolopacidae	*Calidris ferruginea*	Curlew Sandpiper		NT		4
Charadriiformes	Scolopacidae	*Calidris pygmaea*	Spoon-billed Sandpiper	I	CR		8
Charadriiformes	Scolopacidae	*Calidris ruficollis*	Red-necked Stint		NT	1	8
Charadriiformes	Scolopacidae	*Calidris tenuirostris*	Great Knot	II	EN		10
Charadriiformes	Scolopacidae	*Limosa lapponica*	Bar-tailed Godwit		NT		4
Charadriiformes	Scolopacidae	*Limosa limosa*	Black-tailed Godwit		NT		15
Charadriiformes	Scolopacidae	*Numenius arquata*	Eurasian Curlew	II	NT		1
Charadriiformes	Scolopacidae	*Numenius madagascariensis*	Far Eastern Curlew	II	EN		1
Charadriiformes	Scolopacidae	*Numenius phaeopus*	Whimbrel		LC	4	2
Charadriiformes	Scolopacidae	*Tringa brevipes*	Grey-tailed Tattler		NT	2	3
Charadriiformes	Scolopacidae	*Tringa guttifer*	Spotted Greenshank	I	EN		2
Charadriiformes	Scolopacidae	*Tringa nebularia*	Common Greenshank		LC		5
Charadriiformes	Scolopacidae	*Tringa totanus*	Common Redshank		LC	1	1
Charadriiformes	Scolopacidae	*Xenus cinereus*	Terek Sandpiper		LC	9	4
Falconiformes	Falconidae	*Falco peregrinus*	Peregrine Falcon	II	LC		1
Passeriformes	Remizidae	*Remiz pendulinus*	Chinese Penduline Tit		LC	2	
Passeriformes	Acrocephalidae	*Acrocephalus orientalis*	Oriental Reed-Warbler		LC	1	
Passeriformes	Emberizidae	*Emberiza sulphurata*	Yellow Bunting		VU		1
Pelecaniformes	Threskiornithidae	*Platalea minor*	Black-faced Spoonbill	I	EN		40
Pelecaniformes	Ardeidae	*Ardea alba*	Great Egret		LC		1
Pelecaniformes	Pelecanidae	*Pelecanus crispus*	Dalmatian Pelican	I	NT		12

**Table 2:** Number of re-sighting records for species with three or more re-sighting sites.

Species	Migration Connection Regions
*Calidris tenuirostris*	NWA Australia (5), Northern Territory Australia (1), Chongming China (2), Mai Po Hongkong China (1), Kamchatka Russia (1)
*Calidris alpina*	SA Australia (1), Kamchatka Russia (2), Malaysia (1), Jiangsu China (1), Alaska USA (1)
*Calidris canutus*	NWA Australia (2), Chunkotka Russia (1), Bo Bay China (1)
*Calidris ruficollis*	NWA Australia (3), Kamchatka Russia (1), Japan (1), Chongming China (1), Jiangsu China (1)
*Xenus cinereus*	Singapore (1), Japan (1), Mai Po Hongkong China (1), Chongming China (1)
*Calidris pygmaea*	Chunkotka Russia (2), Kamchatka Russia (1), Thailand (2)

## Data Availability

All survey data from this study are included in this published article and its Supplementary Material.

## Supplementary Materials

The following supporting information can be downloaded at: https://www.mdpi.com/article/10.3390/ani14162404/s1, Table S1: List of bird-ringing project in Hangzhou Bay (HZW); Table S2: The recaptured records of bird banding in HZW (2018–2024). # indicated non-local individuals; Table S3: The re-sighting records in Zhejiang from 2021–2024.
